# Prototypes virus of hand, foot and mouth disease infections and severe cases in Gansu, China: a spatial and temporal analysis

**DOI:** 10.1186/s12879-022-07393-4

**Published:** 2022-04-26

**Authors:** Haixia Liu, Yuzhou Zhang, Hong Zhang, Yunhe Zheng, Faxiang Gou, Xiaoting Yang, Yao Cheng, Hannah McClymont, Hui Li, Xinfeng Liu, Wenbiao Hu

**Affiliations:** 1 Division of Infectious Diseases, Gansu Provincial Center for Disease Control and Prevention, Lanzhou, China; 2 School of Public Health and Social Work, Queensland University of Technology, Brisbane, QLD Australia; 3 College of Computer Science and Technology, Zhejiang University, Hangzhou, China; 4 Department of Research, Baolue Technology (Zhejiang) Co., Ltd, Ningbo, China

**Keywords:** Enterovirus 71, Coxsackievirus A16, Hand, Foot and mouth disease, Predicting, Severe cases

## Abstract

**Background:**

Little research has been conducted on the spatio-temporal relationship between the severe cases and the enteroviruses infections of hand, foot and mouth disease (HFMD). This study aimed to investigate epidemic features and spatial clusters of HFMD incidence rates and assess the relationship between Enterovirus 71 (EV71) and Coxsackievirus A16 (CoxA16) and severe cases of HMFD in Gansu province, China.

**Methods:**

Weekly county-specific data on HFMD between 1st January and 31st December 2018 were collected from the China Infectious Disease Information System (CIDIS), including enterovirus type (EV71 and CoxA16), severe and non-severe cases in Gansu province, China. Temporal risk [frequency index (α), duration index (β) and intensity index (γ)] and spatial cluster analysis were used to assess epidemic features and identify high-risk areas for HFMD. Time-series cross-correlation function and regression model were used to explore the relationship between the ratios of two types of viruses (i.e. EV71/Cox16) (EC) and severe cases index (i.e. severe cases/non-severe cases) (SI) of HFMD.

**Results:**

Some counties in Dingxi City, Gansu were identified as a hot spot for the temporal risk indices. Time-series cross-correlation analysis showed that SI was significantly associated with EC (r = 0.417, P < 0.05) over a 4-week time lag. The regression analysis showed that SI was positively associated with EC (*β* = 0.04, 95% confidence interval (CI) 0.02–0.06).

**Conclusion:**

The spatial patterns of HFMD incidence were associated with enteroviruses in Gansu. The research suggested that the EC could be considered a potential early warning sign for predicting severe cases of HFMD in Gansu province.

## Background

Hand, foot and mouth disease (HFMD) is a common childhood disease first reported in New Zealand in 1957, occurring most frequently in children aged under 5 years [1]. HFMD has a global distribution [2–4], and the disease is considered one of the major public health concerns in China. There were 3681 deaths were reported between 2008 and 2020 in China [5, 6]. Following the unprecedented outbreak of HFMD in China in 2008, a national HFMD surveillance system was established in May 2008 [7].

EV71, CoxA16, CoxA6 and CoxA10 are the four main enteroviruses that cause HFMD in China [8]. However, EVA71 and CoxA16 accounted for 67.2% of total cases in the last decade in China [8]. HFMD is caused by infection by viruses from the enterovirus genus, specifically, enterovirus 71 (EV71) and Coxsackievirus A16 (CoxA16) [9]. Several previous studies have shown that EV71 is the main causative agent associated with severe HFMD [10, 11], and is a key risk factor for severe HFMD [12–15]. As a highly neurotropic virus, EV71 is more likely to cause symptoms in the nervous system and develop into severe illness particularly when associated with the fecal–oral transmission route [16]. Severe HFMD cases exhibit severe neurological complications, such as meningitis, encephalitis, cerebrospinal meningitis, pulmonary edema, pulmonary hemorrhage, circulatory disorders, heart damage and other clinical manifestations [17]. EV71 has emerged as the main causative pathogen of HFMD since 1974 and is widely observed in Australia, Europe, Asia and other regions, especially in the Asia Pacific region [18, 19].

Since CoxA16 was first isolated in South Africa in 1951, its prevalence has spread all over the world. CoxA16 and EV71 of different genotypes alternately circulate or co-circulate in East Asia and Southeast Asia, leading to repeated outbreaks of HFMD in this region in the past 20 years [20]. Since the clinical symptoms caused by CoxA16 infection in children are usually mild, there are relatively few studies on the molecular evolution of CoxA16 [21].

However, the current research on severe HFMD incidences considering spatial and temporal distribution is lacking, especially in northwest China. Additionally, the research on the relationship between the HFMD prototype and the severity of cases is limited. Here, we investigated the spatial–temporal patterns in HFMD by enterovirus type and severity of HFMD cases and assessed the relationship between enterovirus types and severe HFMD cases in space and time, which may provide important information of early warning of severe HFMD cases using enterovirus type data.

## Methods

### Study site and data collection

Gansu Province is located in the northwest of China, with an area of 453,700 km^2^, 87 counties/districts and a population of 26.37 million. In 2018, there were outbreaks of HFMD in some regions in Gansu Province, and the number of severe cases was significantly higher than that in previous years [22].

Weekly county-specific data on HFMD between 1st January and 31st December 2018 were collected from the China Infectious Disease Information System (CIDIS). Clinically diagnosed cases and laboratory-confirmed cases of HFMD were collected from CIDIS, where suspected/unknown location data were excluded in the research (36 cases, 0.26%). As EVA71 and CoxA16 predominated enterovirus for HFMD in the last decade in China, and the CIDIS only reported HFMD cases by EV71, CoxA16 and other viruses, we used EV71 and CoxA16 data in the study for analysis. The detailed criteria for clinical and laboratory-confirmed cases and definitions of severe and non-serious cases can be found in The Health Industry Standard of the people's Republic of China (WS 588-2018) [23].

As the guideline of disease surveillance, at least 10 samples of HFMD cases were collected from general hospitals above county level every month, where nucleic acid testing methods were used to identify the virus types as EV71 or CoxA16 [24].

The population data for each county and GDP were obtained from the National Bureau of Statistics [25]. The annual average temperature (AT), annual average precipitation (AP) and GDP at each county, and shapefiles of country boundary were provided by the Chinese Center for Disease Control and Prevention. The implementation of the project has been approved by the ethics committee of Gansu Provincial Centers for Disease Control and Prevention (GSCDCEC2019[012]).

### Data analysis

#### Temporal risk indices analysis

Superimposing different temporal features on geographic indicators are more conducive to identifying spatial risk areas. Therefore, we used three temporal risk indices to assess the degree of epidemic risk: frequency index (α), duration index (β) and intensity index (γ) [26]. The detailed definition and formula for these three indices were demonstrated in our previous work [27].

#### Spatial cluster analysis of temporal risk indices

In order to determine the spatial clustering of three temporal risk indexes of HFMD, we carried out statistics on three temporal risk indices of 87 counties/districts across the province and conducted the spatial cluster analysis through Geoda1.60 software [28, 29]. Spatial visualization and autocorrelation analysis of time risk indicators across the province were performed by ArcGIS 10.2 software (https://www.esri.com/, Esri Inc, Redlands, CA, USA). Moran’s* I* index was used to determine whether the event has spatial autocorrelation. The value range of the global Moran’s *I* is [− 1, 1], standardized statistic Z(I) was used to test statistical significance. When Moran’s *I* > 0 and Z > 1.96, P < 0.05, it indicates that the cases are clustered and there are high-value or low-value aggregation areas; when Moran's *I* < 0 and Z < − 1.96, P < 0.05, This indicates that the cases are discretely distributed; when Moran’s *I* = 0 and the value of Z is between 1.96 and − 1.96, P ≥ 0.05, indicating that the spatial distribution of cases may be random [30].

#### Time-series cross-correlation analysis

The relationship between weekly ratios of EV71/CoxA16 (EC) and weekly severe cases index (severe/non-severe) (SI) at the provincial level and high-risk region were evaluated by time-series cross-correlation analysis [31]. We used the lagged variables with maximum cross-correlation coefficient to develop the models in this paper.

#### Regression analysis

In order to quantify the relationship between EC and SI, we used a linear regression model to assess the relationship between EC and SI after adjusting for AT, AP and GDP [32–34]. This regression analysis was conducted at the provincial level and high infection region, respectively.

$${\text{EC}} = {\text{EV71 cases}}/{\text{CoxA16 cases}}$$

$${\text{SI}} = {\text{severe cases}}/{\text{non - serious cases}}$$

## Results

### Descriptive analysis

A total of 13,571 HFMD cases were reported in Gansu Province between 1st January and 31st December in 2018, ranking second among the notifiable infectious diseases in Gansu Province. From these cases, 1580 patients underwent laboratory tests, including 343 cases of EV71 and 325 cases of CoxA16, 83 of these were classified as severe cases, accounting for 5.25%. In total 8 outbreaks were reported in CIDIS for Gansu province in 2018.

**Fig. 1:**
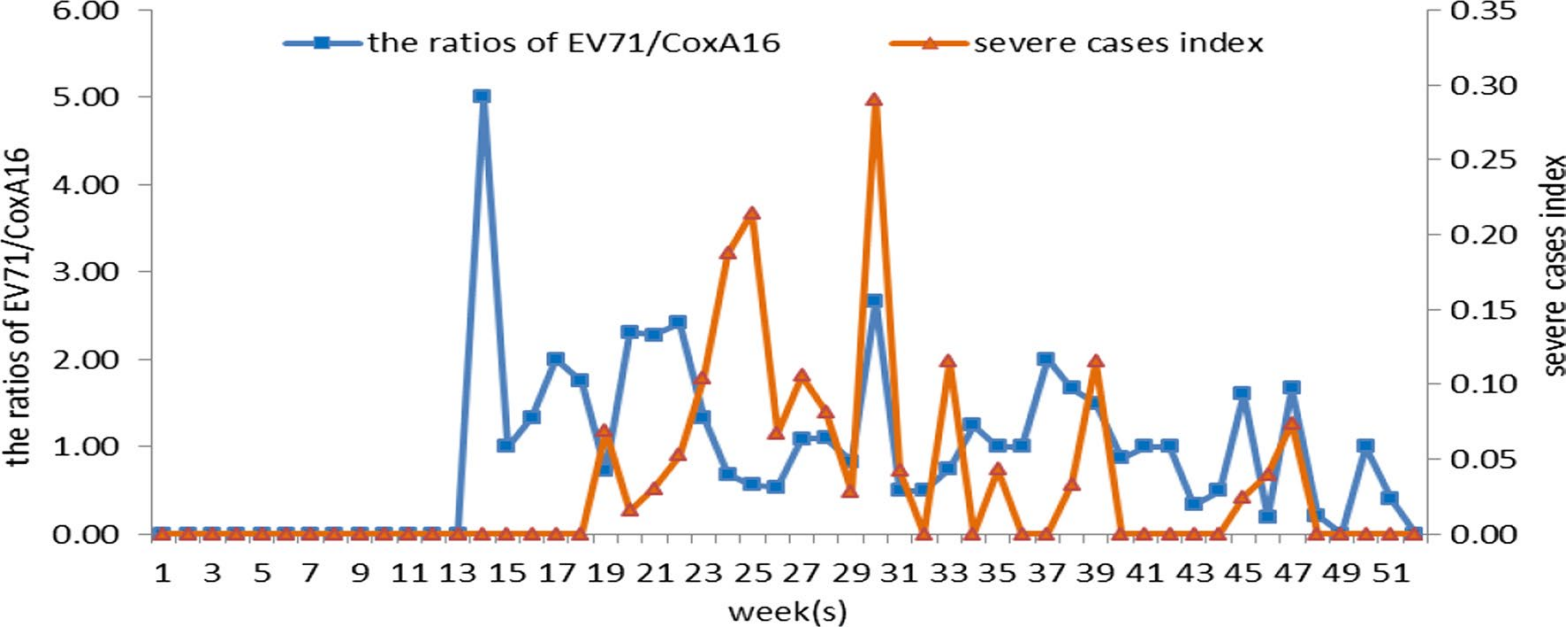
Weekly EC and SI of HFMD in Gansu Province in 2018

The peak incidence of HFMD was from May to July in 2018, accounting for 56.73% of the total number of cases in the whole year, followed by the period from September to November, accounting for 25.19% of all cases. EV71 peaked at 20–28 weeks, CoxA16 peaked at 21–28 weeks, and severe cases concentrated in 23–30 weeks. In 2018, the cases with EV71 were earlier than that of CoxA16 and severe cases. The weekly distribution analysis of EC and SI in Gansu Province showed that EC was earlier than SI in time distribution (Fig. 1).

### Spatial distribution of HFMD

**Fig. 2:**
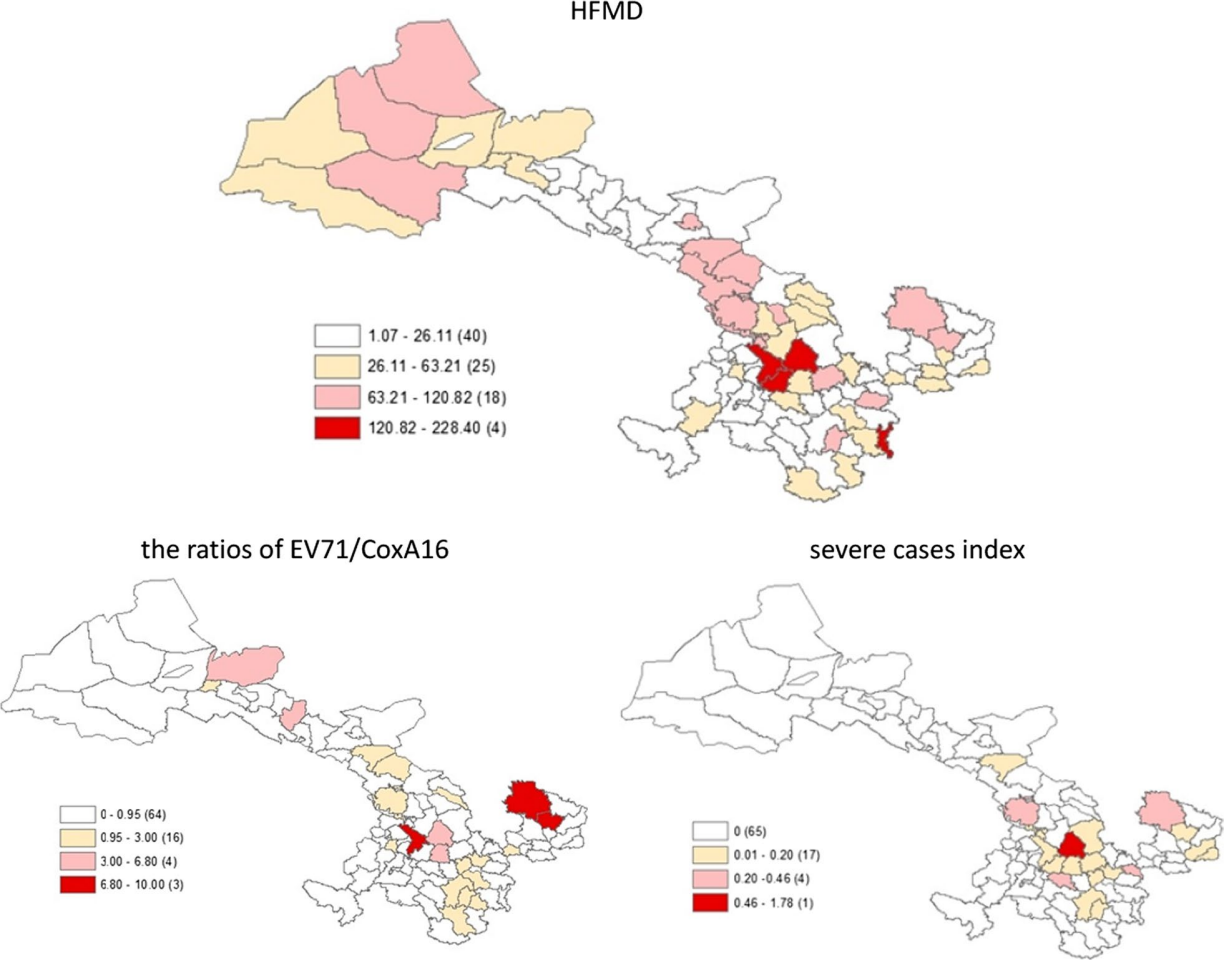
Spatial distribution of virus type incidence of HFMD by EV71 and CoxA16, the ratios of EV71/CoxA16 and severity, EC and SI severe cases index in Gansu Province in 2018

The reported incidence of HFMD in Gansu Province was 51.73/100,000. The areas with a high incidence of EV71 were mainly located in Dingxi City (Lintao County, Anding Qu) and Jiayuguan City. The area with the greatest number of severe HFMD cases was Anding Qu of Dingxi City. According to the regional distribution analysis of EC and SI in Gansu Province, EC was higher in Dingxi City (Lintao County) and Qingyang City (Huan County and Qingcheng County), and SI was higher in Anding Qu of Dingxi City (Fig. 2).

### Spatial pattern analysis of temporal risk indices

The frequency index (α), duration index (β) and intensity index (γ) of EV71 were 0.77, 13.33 and 0.44 separately, and these indices of CoxA16 were 0.79, 13.67 and 0.41 respectively. Spatial autocorrelation analysis showed that Moran’s *I* of the time risk index of EV71 and CoxA16 were both positive (Moran’s *I* > 0, Z > 1.96), indicating that the distribution of EV71 and CoxA16 is clustered and there was a statistically significant positive spatial autocorrelation (Tables 1 and 2).

**Table 1:** Temporal risk indices of EV71 and CoxA16

	CW	TW	PV	IR (/10000)	Frequency index (*α*)	Duration index (*β*)	Intensity index (*γ*)
EV71	40	52	2	1.31	0.77	13.33	0.44
CoxA16	41	52	3	1.24	0.79	13.67	0.41

**Table 2:** Global autocorrelation analysis results of EV71 and CoxA16 temporal risk indices

	Temporal risk indices	Moran’s *I*	P-value	Standard deviance	Z-score
EV71	Frequency index (*α*)	0.15	0.014	0.07	2.40
	Duration index (*β*)	0.20	0.001	0.07	3.05
	Intensity index (*γ*)	0.07	0.012	0.04	2.45
CoxA16	Frequency index (*α*)	0.20	0.002	0.07	3.07
	Duration index (*β*)	0.24	0.001	0.07	3.59
	Intensity index (*γ*)	0.12	0.002	0.04	3.73

**Fig. 3:**
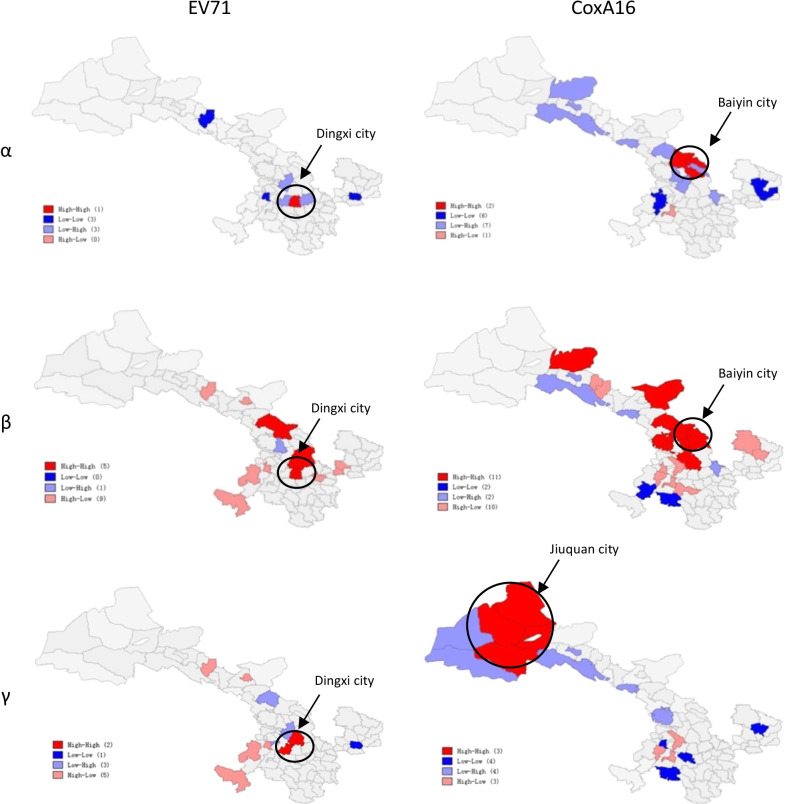
The spatial clusters of EV71 and CoxA16 at temporal risk indices

The local autocorrelation test of temporal risk indices was carried out by using Lisa statistics, indicating that all the high-risk areas of the three temporal risk indices for EV71 were found in the central area of the province (Dingxi city). Moreover, the high-risk areas of the α and β for CoxA16 were observed in the central area of the province (Baiyin city), however, that of γ was observed in the western area of the province (Jiuquan city) (Fig. 3).

### Time series cross-correlation analysis

**Fig. 4:**
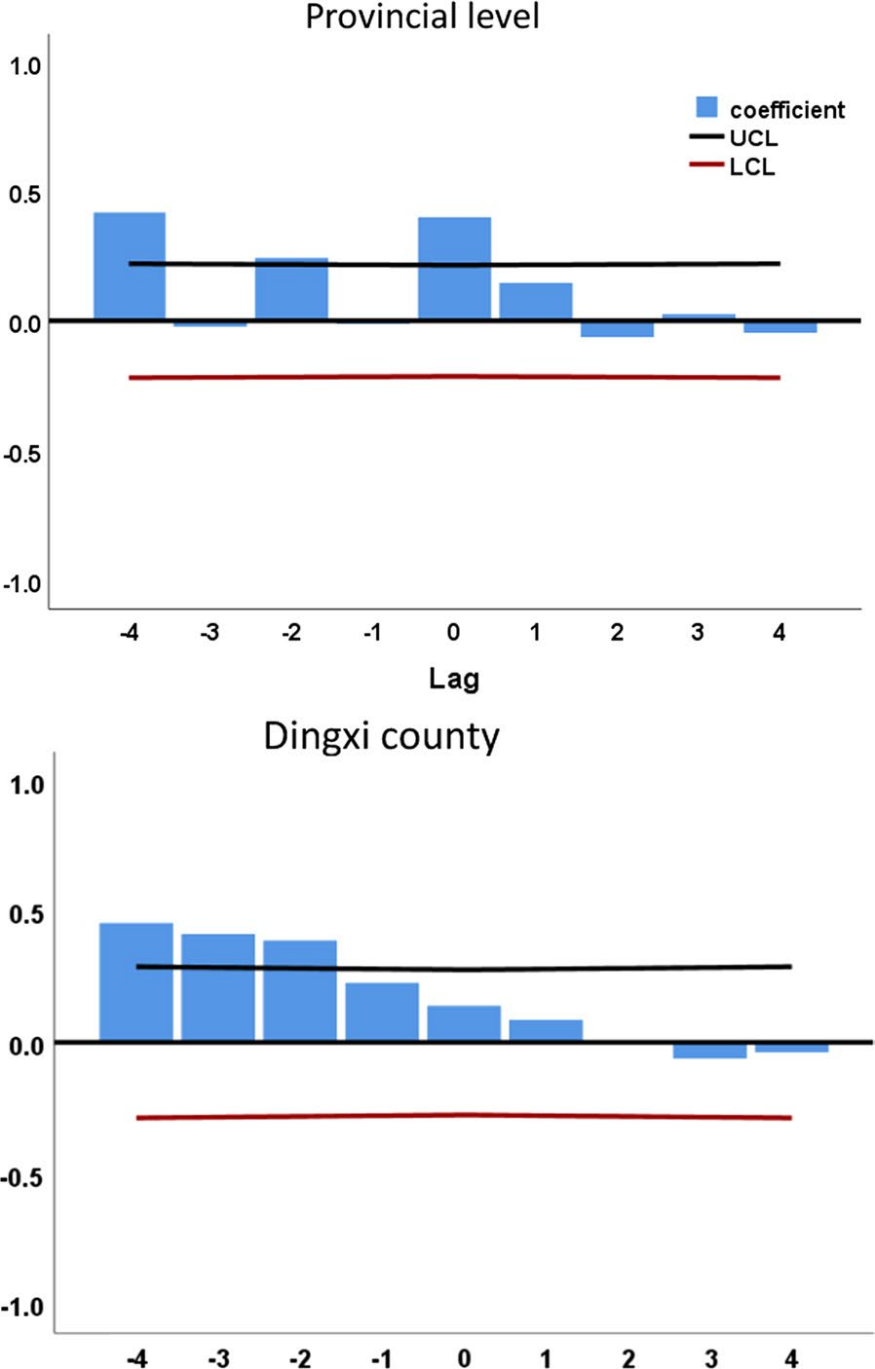
Time series cross-correlation between EC with SI

The time-series cross-correlation analysis demonstrated that EC was strongly correlated with SI at the lag of 4 weeks at both provincial level and high infection area (correlation coefficient of 0.417, P < 0.05) (Fig. 4).

### Regression analysis

**Fig.5:**
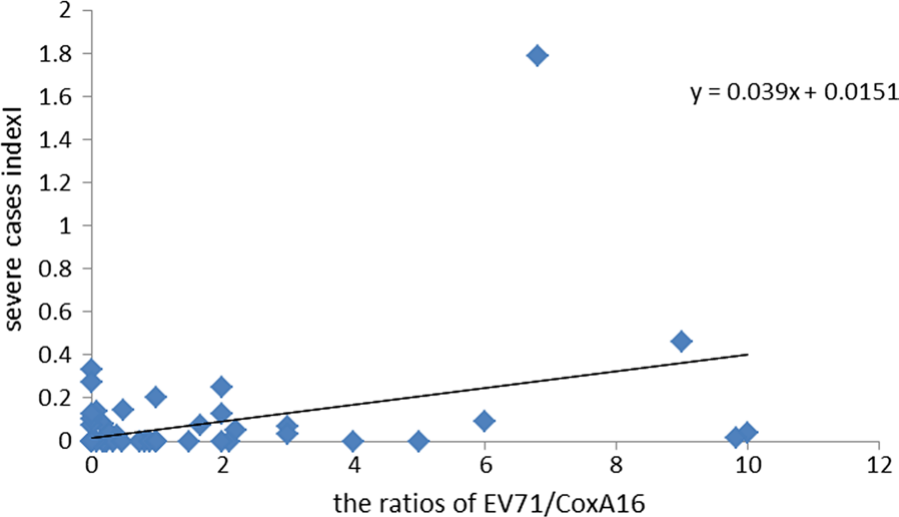
Scatter plots with regression line of EC and SI by county in Gansu Province in 2018

The linear regression model showed that the EC was significantly associated with SI after controlling for AT, AP and GDP variables (β = 0.04, P < 0.0001). Taking into account the influence of extreme values, a sensitivity test was performed on the regression analysis, which confirmed that EC still has an impact on SI (β = 0.039, P = 0.002) (Fig. 5).

**Fig.6:**
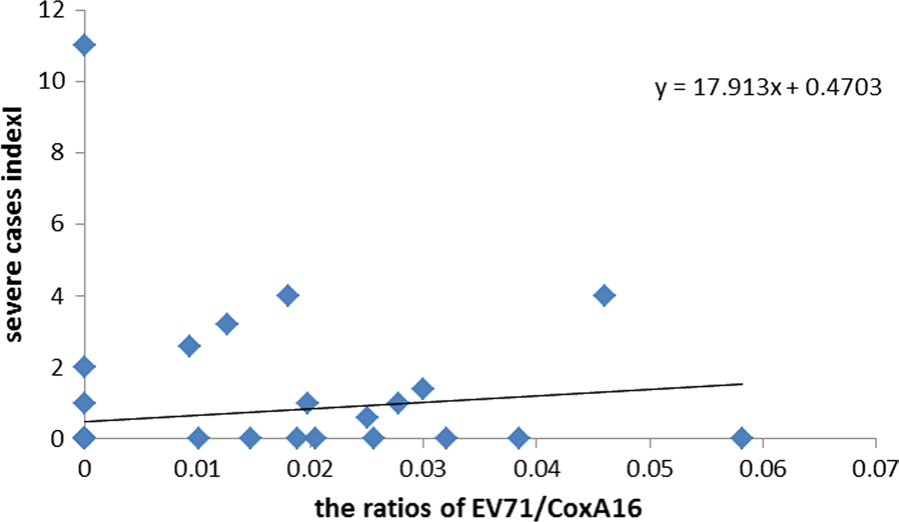
Scatter plots with regression line of weekly of EC and SI in Dingxi city in 2018

In 2018, Dingxi City had 48 severe cases of HFMD, accounting for 57.83% of the province. A total of 6 outbreaks occurred throughout the year. The weekly regression analysis EC had a positive association with SI (Fig. 6).

## Discussion

HFMD is a significant public health concern all over the world [35]. To the best of our knowledge, this is the first study to investigate the relationship between the spatio-temporal distribution of HFMD prototypes and severe cases. EV71 and CoxA16 were the main pathogenic types of HFMD in Gansu Province [6, 36–38]. In this study distribution of EV71 and CoxA16 is clustered and there was a statistically significant positive spatial autocorrelation, the increase in Weekly EC occurred earlier than SI.

EC directly affected the distribution of SI in Gansu province. Therefore, whether the spread of EV71 and CoxA16 could be effectively controlled would be crucial to the occurrence and severe HFMD. In order to monitor the temporal and spatial clustering of the main viral types of HFMD in real-time and predict the epidemic situation of severe HFMD in the whole province, this study analysed the spatial autocorrelation of frequency index, duration index and intensity index of EV71 and CoxA16. The results showed that EV71 had spatiotemporal aggregation in Gansu. Dingxi City rated highest in three temporal risk indices of EV71, and its incidence frequency was high, the duration was prolonged, and transmission intensity was high. This indicates that Dingxi City may be the source of the spread of EV71 in Gansu in 2018 [26]. As a result of this finding, Dingxi City is a key area for HFMD prevention and control. Therefore, it is recommended the relevant health administrative departments should concentrate on health resources and implementing effective measures to control the spread of HFMD in this region. For example, the vaccination coverage for EV71 vaccine should be increased in high-risk areas to improve the herd immunity of the population, if anticipating an increase in EC index; Further strengthening HFMD surveillance and health education are required such as the morning and afternoon inspection of school nurseries and kindergartens at these counties with high EC index. Our research results also provide useful information on supporting clinic workers to care the HFMD patients and reducing the severe cases of HFMD.

EV71 was the most important neurotropic enterovirus after poliovirus eradication [17, 39]. It has become a pathogen causing severe pediatric neurological diseases worldwide, especially in the Asia-Pacific region in recent years [19, 40]. As a highly neurotropic virus, EV71 is transmitted along the nerves and enters the central nervous system through the reverse axonal transport system, leading to nervous system infection [41, 42]. EV71 is most predominant in cases of severe HFMD and associated with higher mortality rates with a positive rate of EV71 testing in over 90% in the fatal cases [5], and it could be considered an independent risk factor for developing severe illness [43]. In this study, the time-series cross-correlation analysis of EC and SI showed that the maximum correlation coefficient occurred 4 weeks ahead of EC, which meant that EC may precede severe cases of HFMD by 4 weeks. This indicated that EC could be seen as an early warning signal for severe cases of HFMD. As this is an ecological study, the results of cross-correlation in this study suggests that there may be a 4-week lag correlation between the proportion of virus types and the proportion of patients with severe diseases. This correlation may not reflect their causal relationship, that is, this correlation cannot make causal inference. However, this lag effect may provide important information in developing early warning for severe cases of HFMD, further research is needed to confirm the biological mechanism between virus type and severe HFMD.

HFMD is a common and frequent acute infectious disease in children, and it takes a certain course for the development of mild cases to severe cases [44]. However, in the township health centres or county-level hospitals, the diagnosis and treatment level of basic medical units is relatively low and laboratory testing assistance is limited. Severe cases of HFMD often need to be referred to designated hospitals at the municipal or provincial level for diagnosis, resulting in a prolonged time interval from the increase in severity of symptoms to diagnosis. Furthermore, due to atypical early rash symptoms of severe cases [45, 46], and clinicians with differing diagnostic and discriminating abilities, misdiagnosis or missed diagnosis may occur [47–49]. Further studies are required to investigate the relationship between the number of EV71 infections and the pathogenicity changes of the EV71 virus. The results of this study showed with the gradual accumulation of cases, the incidence of severe HFMD increased gradually, showing a certain lag in time. Considering that temperature, precipitation and living standard have a certain influence on HFMD [50, 51], after removing these confounding factors in this study, the regression model showed that EC still has a positive impact on SI in space and time dimensions. Regression analysis of Dingxi City with a high proportion of EC and SI also shows that there was a linear positive relationship between EC and SI. Other factors, such as gatherings of people [52, 53] and levels of vaccine coverage [54, 55] may also influence the relationship between EC and SI, which need further investigation in future studies. Furthermore, this may provide scientific evidence for health administrative departments to formulate HFMD prevention, control strategies, and the distribution of health resources when monitoring the patterns of EC.

The key indicators such as EC, SI and temporal risk indices used in this study were mainly derived from the routine monitoring data of HFMD. These indicators developed would allow health professionals to analyse the HFMD data in real-time, thus allowing a timely response. Additional focus should be placed on the areas with high EC values, which may indicate the high possibility of more severe cases. Moreover, timely health education and HFMD surveillance are also required to prevent the transmission of disease and the development of severe cases.

The main limitations identified in this study include the HFMD surveillance system, this is based on a passive reporting system, and bias in reporting information may exist; also, differing levels in clinicians’ knowledge and mastery of diagnostic criteria for HFMD and identifying severe cases may affect reporting and surveillance data [36]. In addition, the CIDIS only reported the HFMD cases by EV71 and CoxA16, other subtypes were not available for this study. Finally, for this study, only the data from Gansu Province over a period of 1 year were analysed, therefore this study cannot determine whether these results differed over the long-term or in other provinces. These are possible considerations for addressing in future research, considering longer study periods and larger-scale research over a greater geographical region for future studies.

## Conclusions

To conclude, our results suggested the spatial patterns of HFMD were correlated with the spatial distribution of enteroviruses in Gansu province in 2018. The temporal index of EC may have the greatest potential as a useful early warning index in predicting severe cases of HFMD, with validation over a wider geographic area with different social and environmental contexts.

## Data Availability

The data that support the findings of this study are available from the China Infectious Disease Information System, but restrictions apply to the availability of these data, which were used under the contract-agreement for the current study, and so are not publicly available. Data are however available from the authors upon reasonable request and with permission of Gansu Provincial CDC.
